# A mixed-methods inquiry into teacher authenticity, psychological capital, identity, and engagement using critical case analysis

**DOI:** 10.3389/fpsyg.2026.1830411

**Published:** 2026-07-14

**Authors:** Lezhi Xiong, Yawen Liu, Zhiqi Wei

**Affiliations:** 1 School of Marxism, China University of Geosciences (Beijing), Beijing, China; 2 School of Marxism, Nanjing University of Science and Technology, Nanjing, China

**Keywords:** critical case analysis, mediation, mixed methods, professional identity, psychological capital, teacher authenticity, teacher well-being, work engagement

## Abstract

**Introduction:**

Grounded in the Job Demands-Resources (JD-R) model, this study investigates the relationships between teacher authenticity, professional identity, psychological capital (PsyCap), and work engagement within mainland China’s educational system, focusing on K-12 teachers (*N* = 627) navigating high-stakes testing environments.

**Methods:**

Employing an explanatory sequential mixed-methods design, the quantitative phase utilized structural equation modeling (SEM) to test a hypothesized mediation model. A subsequent qualitative phase (*N* = 26) involved thematic analysis to corroborate findings and holistic critical case analysis to explain quantitative outliers or “deviant” cases.

**Results:**

Quantitative findings demonstrated that authenticity and professional identity significantly predict work engagement, both directly and indirectly through PsyCap, which emerged as a robust mediator (authenticity total effect *β* = 0.390, *p* < 0.001; professional identity total effect *β* = 0.485, *p* < 0.001). While thematic analysis corroborated these patterns, the critical case analysis revealed that these positive relationships are contingent upon organizational alignment and career stage.

**Discussion:**

These results suggest that while authenticity and identity are vital personal resources, their efficacy in driving engagement is moderated by the institutional context. The study offers nuanced implications for fostering teacher well-being within high-stakes educational environments.

## 1 Introduction

Teacher well-being and effectiveness are widely recognized as foundational to educational quality, yet the profession is increasingly defined by challenges that threaten this foundation. Persistent issues such as high workloads, accountability pressures, and complex classroom dynamics contribute to significant rates of emotional exhaustion, burnout, and attrition globally (Bakker and Demerouti, 2007; Klassen and Chiu, 2010; Sun et al., 2025). In this context, understanding the factors that foster teacher work engagement—a positive psychological state characterized by vigor, dedication, and absorption (Schaufeli et al., 2002)—has become a critical research priority. Engaged teachers are not only more resilient and innovative but also cultivate more positive classroom environments, directly influencing student success (Christian et al., 2011; Wang, 2024; Zee and Koomen, 2016). Therefore, identifying the drivers of engagement is of paramount importance, particularly within demanding educational contexts like mainland China.

Recent research has highlighted the importance of personal resources, such as psychological capital (PsyCap), in promoting teacher work engagement (Avey et al., 2011; Wu, 2025). PsyCap, encompassing self-efficacy, optimism, hope, and resilience (Luthans et al., 2015), has been shown to buffer against stress, enhance job satisfaction, and foster a more positive and engaged approach to work (Guo et al., 2022; Ma, 2023; Zhang et al., 2024). However, the specific antecedents of PsyCap, particularly within the Chinese educational context, remain relatively underexplored. Furthermore, while the relationship between professional identity and work engagement has been investigated (Akkerman and Meijer, 2011; Beijaard et al., 2004), the role of authenticity in this dynamic has received less attention. Authenticity, defined as aligning one’s actions with core values and beliefs (Wood et al., 2008), has been linked to teacher well-being and effective teaching practices (Ramezanzadeh et al., 2017; Sun et al., 2025).

However, a significant theoretical gap exists regarding the cross-cultural transferability of authenticity models. Existing Western frameworks often conceptualize authenticity as an individualistic pursuit of “true self” expression, often positioned in opposition to external social constraints (Cranton and Carusetta, 2004a, 2004b; Wood et al., 2008). In contrast, the Chinese educational landscape is characterized by high power distance and a collectivist ethos that prioritizes relational harmony (*He*) and social duty (*Yiwu*) (Hofstede, 2001). Within this socio-cultural framework, the act of being authentic is not a solitary psychological state but a complex social negotiation. Teachers must navigate the tension between their personal moral core (*Xin*) and the rigid expectations of a hierarchical, high-stakes system (Ball, 2003). This “Harmony-Authenticity Paradox” suggests that Western models may fail to capture how authenticity either builds or depletes psychological resources when individual values clash with collective mandates. Consequently, there is an urgent need for research that investigates how authenticity, professional identity, and PsyCap interact to foster engagement specifically within the nuanced cultural and systemic constraints of mainland China (Sun et al., 2022a; Zhang et al., 2024).

This study addresses these gaps by investigating the intricate relationships between teacher authenticity, professional identity, PsyCap, and work engagement among teachers in mainland China. By employing a rigorous explanatory sequential mixed-methods design, we move beyond conventional data-driven inquiry to explore how these socio-cultural specificities moderate the proposed pathways. The study first aims to quantitatively examine the direct and indirect relationships among these variables, establishing a statistical model of their interplay. Building on these findings, the research then employs a robust qualitative phase designed not only to add depth and context by exploring the common lived experiences of teachers, but also to critically explain the quantitative results. We move beyond simple illustration to probe the *boundaries* of the statistical model, seeking to understand the lived experiences that confirm the general pathways and, crucially, the specific contexts and personal narratives that explain important exceptions to these patterns.

This study fills the identified gaps by providing a nuanced, empirically-grounded model that integrates authenticity, professional identity, and PsyCap as key antecedents of work engagement among Chinese teachers. By integrating the statistical *patterns* from our quantitative phase with the *explanatory power of critical exceptions* from our qualitative phase, the findings will contribute to a deeper and more realistic understanding of teacher motivation and well-being within a culturally specific context, offering valuable implications for interventions aimed at fostering a more engaged and effective teaching workforce in mainland China.

## 2 Theoretical foundations and key constructs

### 2.1 Teacher work engagement: a critical antecedent in China’s evolving educational landscape

Work engagement, a central concept in positive organizational psychology, is a positive, fulfilling state characterized by vigor, dedication, and absorption (Schaufeli et al., 2002). This construct is particularly relevant in the demanding field of teaching. Often conceptualized within the Job Demands-Resources (JD-R) model (Bakker and Demerouti, 2007), engagement is viewed as the antithesis of burnout, marked by energy and active involvement rather than exhaustion (Maslach et al., 2001). In the modern educational landscape, which is increasingly characterized by digital transformation and heightened socio-emotional demands, engagement serves as a critical buffer that allows teachers to thrive despite institutional pressures (Sun et al., 2025; Wang, 2024). In the educational context, vigor signifies stamina for classroom challenges, dedication reflects professional pride, and absorption implies full immersion in the act of teaching (Salanova et al., 2010). These components collectively enable teachers to manage job stress and thrive in difficult school environments (Hakanen et al., 2006; Li et al., 2025).

We engage with the work engagement literature because this construct is central to understanding how teachers survive and flourish under China’s “Double Reduction” reforms and persistent high-stakes examination pressures. While engagement has been extensively studied in Western settings, its drivers in collectivist, high-accountability systems like China’s remain inadequately theorized (Klassen et al., 2012). Recent policy shifts have intensified the need for engaged teachers who can deliver quality education beyond test preparation, yet the systemic pressures that undermine engagement persist (Sun et al., 2022a; Wang, 2024). We review this literature to identify theoretical gaps exposed by China’s unique conditions—specifically, whether personal resources like authenticity and identity operate similarly when relational harmony and duty are prioritized over individual autonomy. Understanding engagement’s antecedents in this context addresses practical concerns about teacher retention and student outcomes in a system undergoing fundamental transformation.

High teacher work engagement yields significant benefits, as it is associated with reduced burnout, greater job satisfaction, and stronger organizational commitment (Klassen and Chiu, 2010; Skaalvik and Skaalvik, 2014). Longitudinal studies highlight its protective effects, showing that engaged teachers report higher efficacy, lower turnover intentions, and less emotional exhaustion, partly through active coping (Bakker and Demerouti, 2007; Hakanen et al., 2006). These advantages extend to pedagogical practice, as highly engaged teachers are more innovative, build stronger student relationships, and demonstrate greater persistence (Klassen et al., 2012; Zee and Koomen, 2016). This link is reinforced by meta-analytic evidence of a reciprocal relationship between engagement and performance, whereby engaged teachers enhance instruction and student achievement (Christian et al., 2011). Thus, work engagement is not merely an individual outcome but a key driver of both teacher well-being and student success.

The antecedents of teacher work engagement involve both personal and organizational resources. Personal resources, such as self-efficacy, optimism, and resilience, foster engagement by building confidence (Bandura, 1997; Luthans et al., 2007a, 2007b; Wu, 2025). Similarly, individual traits like proactivity are linked to higher self-efficacy and satisfaction (Li et al., 2017). Organizational resources are also crucial, including supportive leadership, collaboration, and autonomy, which fulfill needs for competence, relatedness, and independence (Ryan and Deci, 2000; Skaalvik and Skaalvik, 2014; Van den Berghe et al., 2014). Conversely, high job demands like administrative burdens and resource scarcity can drain energy (Bakker and Demerouti, 2007; Klassen and Chiu, 2010; Sun et al., 2025). Emotions also play a role, with positive feelings like joy creating upward spirals of engagement, while negative feelings can deplete it (Burić and Macuka, 2018). While the JD-R model (Bakker and Demerouti, 2007) is a leading framework, it is often complemented by perspectives like Self-Determination Theory (SDT), which suggests that career fit with personal goals is a powerful driver (Timms and Brough, 2013). Such integrated theories provide a fuller picture of teacher well-being, in which engagement often functions as a key mediator (Timms and Brough, 2013; Wang, 2024).

The importance of engagement has spurred various interventions. Job crafting, for instance, allows teachers to modify their tasks to align with personal strengths, fostering ownership (Timms and Brough, 2013). Broader strategies, such as mindfulness and strengths-based development, have also shown success in producing lasting gains (Breso et al., 2011; Dello Russo and Stoykova, 2015), often by balancing demands with strong school supports (Bakker and Demerouti, 2007). Despite this body of research, key limitations remain. Cross-cultural studies show that engagement drivers may differ; in collectivist cultures, shared support might be more influential than individual autonomy (Klassen et al., 2012). Furthermore, the field relies heavily on self-report measures. Consequently, future research has been called to incorporate varied methods, such as observations, for a more robust assessment (Parker et al., 2006).

### 2.2 Teacher psychological capital: a culturally-situated mediator of teacher well-being

Psychological capital (PsyCap), a core construct from positive organizational behavior, functions as a vital personal resource for educators (Luthans et al., 2007a, 2007b). This higher-order resource combines four interconnected components: self-efficacy (confidence in instructional abilities; Bandura, 1997), optimism (positive expectations for professional success; Seligman, 1998), hope (goal-oriented determination and the ability to find routes to goals; Snyder, 2002), and resilience (the capacity to recover from adversity; Masten, 2001). These elements work synergistically, assisting teachers in handling workplace stressors, maintaining commitment, and cultivating positive work experiences (Avey et al., 2011).

Why examine PsyCap in the Chinese context? A central unresolved question concerns the culturally-specific sources that build PsyCap. While its protective and energizing effects are well-documented (Avey et al., 2011; Luthans et al., 2015), its antecedents have been under-theorized, particularly in non-Western settings (Wernsing, 2014). In China, where resilience and optimism may be grounded more in collective belonging and social duty than in individualistic agency, PsyCap may function differently (Guo et al., 2022; Sun et al., 2022b). We engage with this literature to interrogate whether authenticity and professional identity serve as foundational resources cultivating PsyCap—a pathway particularly salient in a culture where moral integrity (Cheng) and role fulfillment (Ze) are central to self-concept. This question carries practical implications for how teacher development programs might be designed in the Chinese system.

Extensive research underscores the role of PsyCap in enhancing teacher well-being. It is consistently linked to lower burnout, stress, and anxiety, and to higher job satisfaction and involvement (Demir, 2018). It appears to function protectively by mitigating negative emotional states (Demir, 2018) and reducing the impact of factors like role stress (Sun et al., 2025). Furthermore, it can combine with other resources, such as authentic leadership, to improve affective well-being (Adil and Kamal, 2016). Within the framework of the JD-R model, PsyCap is increasingly positioned as a robust mediator that facilitates the impact of foundational personal resources on distal work outcomes (Bakker and Demerouti, 2007; Luthans et al., 2007a, 2007b). While some literature explores how engagement might channel the effects of PsyCap, this study aligns with the theoretical stance that PsyCap serves as the psychological mechanism through which deep-seated resources—such as authenticity and professional identity—influence work engagement (Avey et al., 2011; Luthans et al., 2015). For instance, recent investigations in the Chinese context demonstrate that PsyCap mediates the link between internal traits and behavioral manifestations (Ma, 2023), serving as the conduit that translates stable self-perceptions into active vigor and dedication. This recurring mediational role underscores PsyCap’s function in bridging the gap between a teacher’s inner value system and their observable work effort (Guo et al., 2022; Zhang et al., 2024).

However, the sources of this PsyCap are often under-theorized (Luthans et al., 2007a, 2007b; Wernsing, 2014). While most models assume that a “pedagogical identity”—the sense of being a teacher—is the primary driver of a teacher’s resilience and efficacy (Beijaard et al., 2004; Zee and Koomen, 2016), recent work suggests that teacher identity is a “constellation of sub-identities” (Akkerman and Meijer, 2011). For many educators, a “disciplinary” or “subject-matter expert” identity (e.g., seeing oneself as a linguist or scientist who teaches) serves as a distinct psychological anchor (Beijaard et al., 2004; Olsen, 2008). This disciplinary self can provide a sense of mastery and “expert efficacy” that remains resilient even when the teacher’s pedagogical identity is challenged by institutional pressures (Hoyle and John, 1995). In the Chinese context, where the role of the “scholar-teacher” (*xue zhe*) is historically revered, this subject-matter expertise may be an essential, yet overlooked, antecedent to PsyCap (Wei, 2008).

The influence of PsyCap extends to a range of other workplace factors relevant to teaching. It has been positively correlated with workplace spirituality (Wu, 2025) and specific proactive and preventive coping strategies (Mikus and Teoh, 2022). The impact on performance has also been noted, as PsyCap’s role as a mediator ensures that the positive effects of engagement are grounded in psychological endurance (Yao et al., 2022). Some evidence even suggests a reciprocal loop, where work engagement, in turn, strengthens both PsyCap and meaningful work (Tan et al., 2021), reinforcing the extensive reach of PsyCap across teacher experiences.

Although substantial evidence affirms the positive contributions of PsyCap, certain limitations warrant attention. The expression and emphasis of PsyCap components may differ across cultural contexts; for instance, resilience in collectivist societies might be grounded more in social ties than in individualistic optimism (Wernsing, 2014). Moreover, similar to engagement research, the field often depends on self-report measures, introducing risks of common method bias (Podsakoff et al., 2003). Future investigations should therefore integrate diverse methods, such as observational data, for a more objective evaluation. Finally, it remains vital to probe the interactions of PsyCap with systemic elements like leadership and policy (Carmona-Halty et al., 2019) and to use longitudinal designs to track the development of this construct across different career stages.

### 2.3 Teacher professional identity: negotiating the self within China’s educational reforms

Teacher professional identity, a dynamic construct essential for educators, influences both self-perception and practice (Beijaard et al., 2004). It emerges from the continuous integration of personal values with the roles and norms of teaching (Beijaard et al., 2004; Coldron and Smith, 1999; Kelchtermans, 2009). Rather than being fixed, this identity evolves through ongoing negotiation with educational demands (Day and Gu, 2007; Pillen et al., 2013) and is shaped by interactions within broader sociocultural contexts (Hong, 2010; Trent, 2010). Key components generally include subject expertise, didactic competence, and relational agency (Beijaard et al., 2004; Lasky, 2005). Early career experiences are particularly formative, as novice teachers often face “identity dissonance” when their ideals clash with classroom realities (Flores and Day, 2006; Pillen et al., 2013). Structured supports, such as mentoring, reflective practice (Beauchamp and Thomas, 2009), and collaborative professional learning communities (Avalos, 2011; Meijer et al., 2011), are vital for helping beginners align personal ideals with professional constraints to foster a more resilient self-concept.

Professional identity research has global currency, but its application to China requires justification. The Confucian emphasis on role-based identity—where the self is defined through relational obligations—means professional identity in China is not merely a personal construction but a socially negotiated phenomenon (Trent, 2010; Wei, 2008). This is essential because identity formation in China occurs within a web of relationships—to students, parents, administrators, and the state—that Western frameworks often treat as secondary to individual self-definition.

A strong professional identity consistently correlates with positive outcomes, including greater job satisfaction, resilience, and commitment (Day and Gu, 2007; Hong, 2010; Sun et al., 2022a), whereas identity conflicts can lead to exhaustion and attrition (Van Veen and Sleegers, 2006). Well-established identities also link to pedagogical creativity (Hoyle and John, 1995; Zembylas, 2003) and adaptability (Akkerman and Meijer, 2011). Empirical studies confirm that a robust identity promotes career satisfaction, boosts work engagement, and buffers against burnout (Butakor et al., 2021; Jeanson and Michinov, 2020; Li et al., 2025; Sun et al., 2022a; Zhang et al., 2024; Zhai et al., 2023). These relationships are often mediated; work engagement, for instance, frequently mediates the effect of professional identity on career satisfaction and burnout (Jeanson and Michinov, 2020; Li et al., 2025; Zhang et al., 2024; Zhai et al., 2023). Other factors like psychological empowerment, social support, and emotional intelligence also play a role in this complex interplay (Butakor et al., 2021; Li et al., 2025; Sun et al., 2022a; Zhang and Guo, 2023).

Despite these advantages, the construct is not without its critiques. Poststructuralist views, for example, challenge the idea of a stable identity, portraying it as fragmented, fluid, and contested by power dynamics (Clarke, 2009; Zembylas, 2003). Methodological concerns include an over-reliance on self-report measures, which may not capture the full complexity of identity (Beijaard et al., 2004; Canrinus et al., 2012). Addressing these issues requires more nuanced, multifaceted methods. For instance, intersectional approaches are needed to explore how factors like race, gender, and culture affect identity (Klassen et al., 2012; Olsen, 2008; Trent, 2010). Future work should also examine how identity is reshaped by new technologies (Korthagen, 2017; Luehmann, 2007) and track its development longitudinally across entire careers (Beauchamp and Thomas, 2009; Carmona-Halty et al., 2019; Pillen et al., 2013). Incorporating qualitative methods like narrative inquiry can yield richer insights into the lived, dynamic aspects of professional identity (Beauchamp and Thomas, 2009; Pillen et al., 2013).

### 2.4 Teacher authenticity: the harmony-authenticity paradox in the Chinese context

Authenticity, broadly defined as aligning actions with core values and beliefs rather than external pressures (Wood et al., 2008), is increasingly recognized as an essential quality for teacher well-being and performance. Rooted in humanistic psychology, this construct involves self-awareness, unbiased processing of one’s strengths and weaknesses, and behavioral congruence with one’s true self (Kernis and Goldman, 2006; Rogers, 1961; Ryan and Deci, 2000). However, traditional frameworks often emphasize an individualistic “true self” seeking autonomy, a perspective that may overlook the cultural logic of collectivist societies like China, where the “relational self” is paramount (Hofstede, 2001; Lianputtong, 2010).

In the Chinese framework, authenticity is closely tied to the Confucian concept of sincerity (Cheng), which represents an ontological state of moral truthfulness where one’s inner reality and outer manifestation are indistinguishable (Wei, 2008). While Western frameworks often prioritize the discovery of a “true self” in opposition to social roles (Kernis and Goldman, 2006; Rogers, 1961), the Confucian notion of Cheng suggests that the self is realized through the refined performance of those roles. This dictates that moral integrity is verified through the consistent fulfillment of social and professional roles, a concept known as Zhengming (Ramezanzadeh et al., 2017; Wei, 2008). Closely linked to Cheng is the concept of responsibility (Ze), which defines the teacher’s authenticity through their commitment to the moral and intellectual growth of their students (Wei, 2008). Within this cultural logic, Ze is not a restrictive external pressure but a source of volitional strength; a teacher finds their authentic voice by successfully navigating their duty to both the state and the individual student. Thus, for a Chinese teacher, authenticity is not merely personal self-expression; it is a moral obligation to embody the virtues they teach, ensuring that their “inner” character matches their “outer” pedagogical conduct.

We include authenticity to test the cross-cultural transferability of a construct rooted in Western humanistic psychology (Wood et al., 2008; Rogers, 1961). In Western frameworks, authenticity is framed as expressing a “true self” against social constraints. Yet in China, it is better understood through Confucian Cheng (moral sincerity) and Ze (responsibility), where the self is realized through faithful role performance (Wei, 2008; Ramezanzadeh et al., 2017). The “Harmony-Authenticity Paradox” arises because authenticity must coexist with relational harmony (He), creating a tension Western models miss (Ball, 2003; Hofstede, 2001). We ask whether authenticity, understood this way, builds engagement or depends on organizational and cultural alignment—a question made urgent by teachers caught between “authentic pedagogy” rhetoric and performative compliance (Sun et al., 2022a).

In the complex setting of education, authenticity requires teachers to continuously negotiate institutional requirements, such as standardized curricula, with their personal teaching philosophies (Cranton, 2006; Gu and Day, 2013; Kreber et al., 2007; Sutton, 2020). Within Chinese education, this requires navigating the tension between moral sincerity and a systemic “performativity” (Ball, 2003) driven by high-stakes testing and rigid administrative hierarchies. Nowadays, this tension has taken on a digital and systemic dimension. While the “Double Reduction” reforms aimed to ease academic pressure, they shifted the burden toward “Quality Education” (*Su Zhi Jiao Yu*), requiring teachers to be more emotionally present and “authentic” in their pedagogy (Sun et al., 2022a). However, this mandate often clashes with the reality of digital performativity. With the ubiquity of school-parent communication platforms and AI-driven teacher evaluation metrics, the boundary between a teacher’s private “authentic” self and their public “harmonious” persona has largely collapsed (Lasky, 2005; Van Veen and Sleegers, 2006). Consequently, social harmony (*He*) is no longer just a cultural preference but an institutional requirement enforced by constant visibility. This shapes teacher behavior into a form of “negotiated integrity,” where teachers must carefully calibrate their authentic professional values against a state-sanctioned version of “duty” that is increasingly monitored in real-time. Students, in turn, often perceive authenticity as accessibility, passion, and commitment, specifically viewing it through the lens of a teacher’s moral consistency and “heart” (*xin kan*). This fosters a trusting environment linked to student motivation and prosocial actions (Cranton and Carusetta, 2004a, 2004b; De Bruyckere and Kirschner, 2016; Leroy et al., 2015). In this context, the enactment of authenticity becomes an act of “pedagogical courage”—the willingness to maintain value-driven instruction despite intense institutional pressures for exam-oriented results (Trent, 2010).

Teachers themselves tend to describe authenticity not as a static trait but as a dynamic “ongoing journey” (Malm, 2008) or a “becoming” process, grounded in self-reflection, hope, and relational insight (Cranton and Carusetta, 2004a, 2004b; Ramezanzadeh et al., 2017). The ability to enact authenticity is influenced by both individual and organizational factors. It is supported by individual traits like reflective commitment and moral courage (Day and Gu, 2007; Kreber et al., 2007) as well as by supportive organizational elements, including autonomy and collaborative cultures (Pearson and Moomaw, 2005). Conversely, rigid accountability pressures can stifle it, forcing teachers into “survival strategies” of compliance over real engagement (Ball, 2003; Kelchtermans, 2009). The consequences are significant, as authentic teachers report greater psychological health and purpose derived from value-aligned work (Ryan and Deci, 2000; Sutton, 2020). This alignment also drives proactive behaviors, such as innovation and resilience (Hong, 2010; Lianputtong, 2010), and can extend to authentic leadership behaviors that enhance team efficacy (Avolio et al., 2004; Leroy et al., 2015). A lack of authenticity, conversely, is associated with burnout and attrition (Hong, 2010).

Investigating authenticity, professional identity, and PsyCap as a unified “resource bundle” allows for a multi-dimensional view of the teacher as a moral, social, and psychological agent (Luthans et al., 2015; Ramezanzadeh et al., 2017). Within the motivational process of the Job Demands-Resources (JD-R) model, these constructs function synergistically to sustain engagement (Bakker and Demerouti, 2007; Hakanen et al., 2006). Authenticity serves as the “moral core,” ensuring that energy is derived from genuine value-congruence rather than surface-level compliance (Cranton and Carusetta, 2004a, 2004b; Malm, 2008). Professional identity provides the “social framework,” defining the teacher’s role and sense of belonging (Beijaard et al., 2004; Pillen et al., 2013). Finally, PsyCap acts as the “agentic engine,” providing the psychological resources—hope, efficacy, resilience, and optimism—necessary to translate these values and roles into persistent effort (Luthans et al., 2007a, 2007b). By examining these constructs in tandem, this study moves beyond fragmented trait analysis to explore how internal convictions and social self-concept are converted into visible work engagement (Guo et al., 2022; Sun et al., 2022b).

Despite this growing body of evidence, the construct is not without conceptual and methodological critiques. Poststructuralist perspectives challenge the notion of a single “true self,” viewing identity as fluid, context-bound, and shaped by societal discourse (Clarke, 2009; Zembylas, 2003). Methodologically, quantitative studies often face measurement flaws, including difficulty in separating authenticity from related constructs like autonomy (Kernis and Goldman, 2006) and a heavy reliance on self-report data (Podsakoff et al., 2003). Cross-cultural differences are also a critical consideration; authenticity in collectivist societies, which value harmony and duty, may be expressed differently than in individualistic ones (Hofstede, 2001). For instance, research in Hong Kong found that teachers linked authenticity more to fulfilling responsibilities and moral mandates than to personal self-expression (Trent, 2010). New contexts, such as the integration of AI in education, also raise fresh questions about the construct (Korthagen, 2017).

To deepen the field’s understanding, future research should employ more complex methods. This includes using intersectional approaches to explore how authenticity intersects with race, gender, and class (Crenshaw, 1991; Lianputtong, 2010). Longitudinal and mixed-methods designs are also necessary to clarify how authenticity evolves across a teacher’s career and in response to policy shifts (Day and Gu, 2007; Gu and Day, 2013). Finally, there is a need to evaluate specific interventions, such as mindfulness and narrative reflection, that aim to cultivate authenticity and its associated benefits (Rodgers and Scott, 2008). Tackling these issues can yield a more robust, culturally responsive grasp of authenticity as a driver of effective teaching.

### 2.5 The purpose of the study

This study aims to delve into the intricate interplay of teacher authenticity, professional identity, psychological capital (PsyCap), and work engagement within the specific context of mainland China’s educational system. By doing so, it addresses a significant theoretical gap: the degree to which Western-derived models of authenticity apply to an educational landscape defined by Confucian values and top-down institutional control. The rationale for this focus is twofold. First, China’s current transition toward “Quality Education” (*Su Zhi Jiao Yu*) demands teachers who are more than mere conduits of information, yet the system’s structural rigidity often penalizes the very “authentic” behaviors it claims to encourage. Second, we examine whether authenticity in a relational culture serves as a different kind of psychological resource—one based on social duty (Ze) and moral sincerity (Cheng) rather than individualistic autonomy.

This study contributes to theory by integrating work engagement, psychological capital, professional identity, and authenticity within a unified model for China’s context. Although these constructs have been studied separately (Akkerman and Meijer, 2011; Beijaard et al., 2004), their interplay—particularly PsyCap’s mediating role and the moderating effects of culture and organization—has not been systematically examined. Our contribution lies in engaging directly with China’s socio-cultural and policy landscape. The “Double Reduction” reforms, digital performativity, and Confucian values are central to understanding how these relationships operate (Hofstede, 2001; Wernsing, 2014). We challenge the universal applicability of Western individualistic models, proposing instead a culturally-situated framework where authenticity and identity are socially negotiated, PsyCap is culturally contingent, and engagement hinges on organizational fit and career stage. Methodologically, our explanatory sequential mixed-methods design with critical case analysis addresses a gap: most studies use either quantitative or qualitative approaches alone, missing the explanatory power of exceptions (Creswell and Plano Clark, 2017). By analyzing “deviant” cases, we probe the model’s boundaries—revealing when and why relationships deviate—producing both confirmatory evidence and theoretical refinement.

Grounded in the existing literature and the need to understand the nuanced dynamics of teacher motivation, this research tests a conceptual model centered on the following five hypotheses.

Authenticity, defined as acting in congruence with core values, is theorized to promote a sense of purpose and fulfillment. Research suggests that when teachers align their pedagogy with their internal value systems, they demonstrate higher levels of professional vigor and dedication (Cranton and Carusetta, 2004a, 2004b; Ramezanzadeh et al., 2017; Wood et al., 2008). Based on this logical derivation:

*Hypothesis 1 (H1)*: Teacher authenticity is positively related to work engagement.

Similarly, a robust professional identity serves as a psychological anchor that enhances job satisfaction and commitment. When teachers view their role as a calling, they are more likely to exhibit sustained work engagement (Beijaard et al., 2004; Day and Gu, 2007; Sun et al., 2022a). Consequently:

*Hypothesis 2 (H2)*: Teacher professional identity is positively related to work engagement.

As a higher-order personal resource, PsyCap provides the cognitive-affective energy necessary for teachers to maintain a positive outlook and navigate workplace stressors effectively (Guo et al., 2022; Luthans et al., 2007a, 2007b; Ma, 2023). Therefore:

*Hypothesis 3 (H3)*: Psychological capital (PsyCap) is positively related to work engagement.

Within the resource bundle framework, it is posited that authenticity facilitates the accumulation of PsyCap by reducing cognitive dissonance and fostering genuine self-reflection. This moral resource is expected to strengthen the “agentic engine” of the teacher, which subsequently fuels higher engagement. Thus:

*Hypothesis 4 (H4)*: PsyCap mediates the relationship between teacher authenticity and work engagement.

Furthermore, a strong professional identity provides a social framework that cultivates internal states of hope and resilience. This suggests that identity builds the psychological capacity required for active work involvement. We therefore propose:

*Hypothesis 5 (H5)*: PsyCap mediates the relationship between teacher professional identity and work engagement.

In addition to these quantitative hypotheses, this study aims to qualitatively explore the lived experiences of teachers. A specific objective of this phase is to probe the “plurality” of identity—exploring how different sources of self (e.g., pedagogical vs. disciplinary) contribute to a teacher’s psychological resources. More critically, this phase aims to move beyond general themes to explain the quantitative model’s complexities. We specifically seek to analyze “deviant” or “critical” cases—defined here as individuals whose experiences contradict hypothesized patterns. These include “conflicted authentic” teachers (high authenticity but low engagement), “engaged but inauthentic” teachers (low authenticity but high engagement), and “resilient but detached” teachers (high PsyCap but low professional identity). By integrating quantitative correlations and qualitative exceptions, this study seeks to provide a comprehensive and realistically nuanced understanding of teacher motivation within the specific context of mainland China’s educational system.

## 3 Method and materials

This study adopted a rigorous explanatory sequential mixed methods design, commencing with a robust quantitative phase to quantify the relationships among authenticity, teacher professional identity, psychological capital, and work engagement among teachers in mainland China. Subsequently, the quantitative findings were elaborated upon in a qualitative phase, employing semi-structured interviews to elucidate in-depth explanations and provide richer, contextualized insights into the quantitative results (Creswell and Plano Clark, 2017). This methodologically triangulated approach facilitated a comprehensive and nuanced understanding of the proposed model, integrating both the statistical breadth of large-scale patterns and the interpretive depth of teachers’ lived experiences.

### 3.1 Participants and setting

Participants in the quantitative phase consisted of 627 teachers (314 female, 313 male) strategically recruited from a heterogeneous array of educational contexts across mainland China. This encompassed a spectrum of institutions: primary schools (38.9%, *N* = 244), secondary schools (43.2%, *N* = 271), and vocational institutions (17.9%, *N* = 112). To maximize the representativeness of the sample and bolster the generalizability of the findings to the broader teacher population in mainland China, a stratified random sampling technique was implemented. This methodologically sound approach deliberately oversampled from predefined strata based on school types and established geographical regions, specifically targeting East (31.6%, *N* = 198), Central (35.7%, *N* = 224), and West China (32.7%, *N* = 205) regions. Out of 780 questionnaires initially distributed to the participating schools, 695 were returned. Following a rigorous screening process to exclude incomplete responses or those exhibiting systematic bias (e.g., straight-lining), 627 cases were retained for the final analysis, yielding an effective response rate of 80.4%. Detailed demographic data indicated that participants possessed a considerable range of teaching experience, spanning from 2 to 25 years (Mea*N* = 9.7 years, SD = 5.3 years), and their ages ranged from 25 to 50 years (Mea*N* = 35.2 years, SD = 6.8 years). Participation was entirely volitional, and formal documentation of informed consent was meticulously obtained from each participant prior to the inception of data collection under the oversight of the Institutional Review Board (IRB) of the China University of Geosciences (Approval No. 2025-CUG-042).

In the ensuing qualitative phase, a carefully selected subset of 26 teachers was purposively drawn from the initial quantitative sample. This sample size was judged sufficient based on the principle of data saturation, whereby thematic redundancy was anticipated and the point of diminishing returns for new substantive insights was projected to be reached within this number of interviews (Braun and Clarke, 2006). The selection strategy prioritized capturing a maximum variation sample, purposefully representing a range of scores from the quantitative measures. This involved targeted recruitment of teachers exhibiting high, medium, and low levels of work engagement and psychological capital to ensure a diverse array of perspectives. Crucially, this maximum variation sampling was also instrumental for identifying critical cases—individuals whose quantitative scores represented combinations that challenged or deviated from the main hypothesized model (e.g., high authenticity but low work engagement, or high PsyCap but low professional identity). This purposeful inclusion of statistical outliers allowed for a deeper explanatory analysis beyond simple confirmation. The qualitative sample comprised 15 females and 11 males, with a mean age of 38.1 years (SD = 7.2 years) and an average teaching experience of 11.4 years (SD = 5.9 years), demonstrating a broadly comparable demographic profile to the larger quantitative sample.

### 3.2 Instruments

Data for the quantitative phase were collected using validated self-report questionnaires with well-established psychometric properties in prior research. Each instrument used Likert-type scales. To ensure linguistic and conceptual equivalence between the original English instruments (AS, PCQ-24, and UWES-17) and the Chinese versions used in this study, a rigorous back-translation procedure was followed (Brislin, 1970). First, two bilingual researchers independently translated the scales into Chinese. Second, another bilingual academic, who was blinded to the original English versions, back-translated these drafts into English. Finally, a panel of three experts in Applied Linguistics and Psychology compared the back-translated versions with the originals to resolve any discrepancies in nuance or cultural connotation, ensuring that the Chinese items accurately captured the intended constructs within the local educational context. In this study, internal consistency for each scale was assessed using Cronbach’s *α*, with values indicating good reliability in the current Chinese teacher sample. Details on each instrument follow.

#### 3.2.1 Authenticity scale

Participants completed the 12-item Authenticity Scale (AS) to assess authenticity (Wood et al., 2008). This scale defines authenticity through three subscales: Authentic Living (behavior matching core beliefs), Self-Alienation (lack of self-understanding, reverse-scored), and Acceptance of External Influence (susceptibility to pressures, reverse-scored). Sample items include “I always know what I want” (Authentic Living), “I do not know how I really feel inside” (Self-Alienation, reverse-scored), and “I often do things based on what other people expect of me” (Acceptance of External Influence, reverse-scored). Items were rated on a 7-point scale from 1 (Strongly disagree) to 7 (Strongly agree). Higher scores indicate greater authenticity, with overall and subscale options available. The AS has shown strong internal consistency and validity in past evaluations (Wood et al., 2008). In this sample, the overall scale had good reliability (Cronbach’s *α* = 0.84), with subscale values of 0.81 for Authentic Living, 0.79 for Self-Alienation, and 0.76 for Acceptance of External Influence.

#### 3.2.2 Teachers’ professional identity scale

Teacher professional identity was measured with the 18-item Teachers’ Professional Identity Scale (TPIS), adapted from Wei (2008). It covers four areas: occupational values, teaching role value, sense of belonging in the profession, and professional conduct inclination. Examples include “Teaching is a career that aligns with my personal values” (occupational values), “I am clear about the responsibilities of a teacher” (role value), “I feel like I am part of the teaching profession” (sense of belonging), and “I actively participate in professional development activities” (professional behavior inclination). Responses used a 5-point scale from 1 (Very strongly disagree) to 5 (Very strongly agree). Although the instrument has a long-standing history in regional research, it was selected because it targets the foundational moral and relational anchors of the teaching profession that remain stable despite structural educational reforms. To ensure the scale’s continued relevance for the present study’s timeframe, its factor structure was empirically re-examined. As reported in the measurement model results (Section 4.1.2), the four-factor structure demonstrated high construct validity and strong fit indices with the current sample, confirming that the items effectively capture the psychological realities of modern educators. In this sample, it had solid internal consistency (Cronbach’s *α* = 0.89), with subscales ranging from 0.82 to 0.87.

#### 3.2.3 Psychological capital questionnaire

Psychological capital (PsyCap) was assessed with the 24-item Psychological Capital Questionnaire (PCQ-24; Luthans et al., 2015). It evaluates four dimensions: Self-Efficacy, Hope, Resilience, and Optimism, with six items each. Samples include “I feel confident analyzing a long-term problem to find a solution” (Self-Efficacy), “I can think of many ways to reach my current work goals” (Hope), “When I have a setback at work, I recover quickly” (Resilience), and “I always look on the bright side of things regarding my job” (Optimism). Ratings were on a 6-point scale from 1 (Strongly disagree) to 6 (Strongly agree). Higher total and subscale scores mean greater PsyCap. The PCQ-24 has proven validity across settings and cultures (Luthans et al., 2015). In this sample, the overall scale showed high reliability (Cronbach’s *α* = 0.92), with subscales at 0.88 for Self-Efficacy, 0.85 for Hope, 0.87 for Resilience, and 0.84 for Optimism.

#### 3.2.4 Utrecht work engagement scale

Work engagement was measured with the 17-item Utrecht Work Engagement Scale (UWES-17; Schaufeli and Bakker, 2003). It assesses three dimensions: Vigor (high energy and resilience), Dedication (involvement and enthusiasm), and Absorption (immersion in work). Items include “At my work, I feel bursting with energy” (Vigor), “I am enthusiastic about my job” (Dedication), and “When I am working, I forget everything else around me” (Absorption). Responses used a 7-point scale from 0 (Never) to 6 (Every day). Higher total and subscale scores indicate more engagement. The UWES-17 has strong validity, consistency, and stability (Schaufeli and Bakker, 2003). In this study, it had good reliability (Cronbach’s *α* = 0.90), with subscales at 0.86 for Vigor, 0.88 for Dedication, and 0.84 for Absorption.

#### 3.2.5 Semi-structured interviews

Following the quantitative data analysis, a qualitative phase was rigorously implemented to provide in-depth, contextualized explanations for the statistical relationships observed in the quantitative data. Semi-structured interviews were conducted with 26 teachers, strategically and purposefully selected to represent a spectrum of experiences and perspectives related to the study variables. The interview protocol was meticulously developed, directly informed by the quantitative findings and the underlying theoretical framework of the research model. Employing open-ended, probing questions, the interviews aimed to elicit rich, descriptive data regarding teachers’ subjective understandings and lived experiences of authenticity in their professional roles, their articulation of professional identity, their access to and utilization of psychological resources, and the complex, nuanced ways in which these factors interacted to collectively shape their work engagement. Exemplar questions included: “Could you elaborate on what it means for you to embody ‘authenticity’ in your daily work as a teacher?,” “How would you characterize your professional identity as a teacher, and which elements are most salient or defining for you?,” “Reflecting on your teaching career, can you recount specific instances where your psychological strengths have been particularly instrumental in navigating professional challenges or enhancing your teaching effectiveness?,” and “What specific aspects of your teaching role do you find most intrinsically engaging, and what factors contribute to these feelings of engagement?”

Each interview, lasting between 45 and 60 min, was audio-recorded to ensure fidelity in capturing verbal data and subsequently transcribed verbatim in the original Chinese to facilitate systematic and rigorous thematic analysis. To ensure the linguistic and conceptual integrity of the qualitative data, a rigorous three-step translation protocol was implemented. Initially, the primary researcher translated key excerpts and codes into English. Subsequently, a bilingual academic back-translated these materials into Chinese to identify potential shifts in meaning (Brislin, 1970). Any discrepancies regarding cultural nuances or professional terminology were then resolved through collaborative consensus. To further enhance the trustworthiness and credibility of the findings, member checking was employed (Lincoln and Guba, 1985). Participants were provided with summaries of their interview transcripts and preliminary thematic interpretations, enabling them to verify the accuracy of the data and ensure that the findings authentically represented their perspectives.

### 3.3 Data collection procedures

Prior to the commencement of data collection, formal ethical clearance was diligently secured from the China University of Geosciences (IRB Approval No. 2025-CUG-042), ensuring adherence to all relevant ethical guidelines and principles for research involving human subjects. Participant recruitment for the quantitative phase was proactively facilitated through established institutional collaborations with regional education authorities and direct engagement with school administrators across mainland China. To optimize response rates while minimizing disruption to instructional time, questionnaires were administered in a paper-and-pencil format during pre-scheduled staff meetings or professional development sessions. A carefully trained team of research assistants was present during these sessions to provide standardized instructions, address participant inquiries, and rigorously uphold the confidentiality and anonymity of all responses.

For the qualitative phase, teachers from the quantitative sample who had previously expressed their willingness to participate in follow-up interviews were individually contacted. Interviews were intentionally conducted in private, comfortable settings, specifically at the participants’ respective schools in designated quiet rooms or at an alternative mutually agreed upon location, to foster an environment optimally conducive to open, honest, and in-depth dialogue. Prior to each interview, participants were explicitly and verbally reassured regarding the strict confidentiality of their responses and were re-affirmed of their voluntary participation, including the unqualified right to withdraw at any point without consequence, thereby reinforcing the ethical imperatives of voluntary participation and informed consent.

### 3.4 Data analysis

Quantitative data were analyzed with SPSS Version 27. Initial steps included computing descriptive statistics for all study variables to outline sample features and variable distributions. To address potential Common Method Bias (CMB) inherent in the self-reported, cross-sectional design, both procedural and statistical remedies were applied. Procedurally, participants were assured of total anonymity and confidentiality to reduce social desirability bias, and survey items were refined for clarity to minimize ambiguity. Statistically, Harman’s single-factor test was employed to assess whether a single dominant factor accounted for the majority of the variance in the data. Bivariate links among authenticity, teacher professional identity, psychological capital, and work engagement were examined via Pearson correlation analysis. To test the mediation model rigorously, Structural Equation Modeling (SEM) followed in AMOS Version 26. SEM allowed simultaneous evaluation of direct and indirect paths, including psychological capital’s mediation in the ties between authenticity and teacher professional identity with work engagement. Model fit was assessed using standard indices: Chi-square statistic, Comparative Fit Index (CFI), Tucker-Lewis Index (TLI), Root Mean Square Error of Approximation (RMSEA), and Standardized Root Mean Square Residual (SRMR). Fit was considered acceptable based on common thresholds (Hu and Bentler, 1999).

Qualitative data, consisting of verbatim interview transcripts, underwent a two-stage analysis process. First, a foundational thematic analysis was conducted following Braun and Clarke's (2006) guidelines to identify broad, recurring patterns across the entire qualitative sample. The process was iterative and multi-staged: (1) Transcription and familiarization; (2) Initial inductive coding to identify key meanings; (3) Theme development by organizing codes into broader patterns; (4) Theme refinement and review; and (5) Final theme definition and naming. This stage provided a comprehensive overview of the common ways teachers experienced the core constructs.

Second, to enhance the explanatory power of the study, we conducted a holistic critical case analysis (Patton, 2015). This stage moved beyond identifying common themes to explaining quantitative anomalies. We purposefully selected five “deviant” cases from the 26-teacher sample based on specific statistical criteria: these individuals exhibited standardized residuals exceeding ±2.0 standard deviations from the predicted regression paths in the SEM model. Specifically, we targeted “asymmetric” cases where high scores in antecedent variables (e.g., Authenticity or Professional Identity) resulted in unexpectedly low engagement scores, or vice versa. These cases were not broken apart into themes but were analyzed holistically, treating each teacher’s transcript, narrative, and survey scores as a complete and unique case. The analysis focused on identifying idiosyncratic factors, personal histories, and specific school contexts that could explain *why* their experience diverged from the model’s predictions. From this holistic analysis of all five cases, the three most conceptually distinct and representative examples were selected for detailed presentation in the findings section to illustrate the different ways the model’s boundaries were challenged.

For analysis trustworthiness, inter-coder reliability for the initial thematic analysis was established using Cohen’s kappa: two researchers coded 25% of transcripts independently, achieving an average kappa of 0.82 (ranging from 0.78 to 0.86 across themes), indicating substantial agreement; differences were resolved through discussion and consensus. The critical case analyses were developed by the primary researcher and then audited by a second team member to ensure the narrative interpretations were “grounded in the data” (Corbin and Strauss, 2008) and plausibly integrated with the quantitative scores.

## 4 Findings

### 4.1 Quantitative results

#### 4.1.1 Descriptive statistics

Descriptive statistics were calculated for the main study variables: Authenticity, Teacher Professional Identity, Psychological Capital (PsyCap), and Work Engagement. Table 1 presents means, standard deviations, ranges, skewness, and kurtosis values. All scales showed suitable means and standard deviations, reflecting a balanced score distribution in the sample. Skewness and kurtosis fell within acceptable limits (±1.0), indicating that the data closely followed a normal distribution, meeting a core assumption for later analyses.

**Table 1:** Descriptive statistics for study variables (*N* = 627).

Variable	Mean	SD	Range	Skewness	Kurtosis
Authenticity (AS)	5.21	0.85	1.00–7.00	−0.32	0.15
Teacher professional identity (TPIS)	3.95	0.62	1.00–5.00	−0.18	−0.05
Psychological capital (PCQ-24)	4.12	0.70	1.00–6.00	−0.25	0.08
Work engagement (UWES-17)	4.38	0.91	0.00–6.00	−0.41	0.22

AS, Authenticity Scale; TPIS, Teachers’ Professional Identity Scale; PCQ-24, Psychological Capital Questionnaire-24; UWES-17, Utrecht Work Engagement Scale-17.

Before proceeding to hypothesis testing, Harman’s single-factor test was conducted using Exploratory Factor Analysis (EFA) with all scale items to assess potential Common Method Bias (CMB). The results revealed that the first emerging factor accounted for only 29.4% of the total variance. Since this value is well below the 50% threshold typically cited in the literature (Podsakoff et al., 2003), it suggests that common method variance does not pose a significant threat to the validity of the findings.

Before examining the mediation model, bivariate correlations were assessed with Pearson’s correlation coefficient (*r*). Table 2 displays these results, with all expected relationships significant and positive. Authenticity correlated positively with Teacher Professional Identity (*r* = 0.45, *p* < 0.001), Psychological Capital (*r* = 0.52, *p* < 0.001), and Work Engagement (*r* = 0.48, *p* < 0.001). Teacher Professional Identity showed positive correlations with Psychological Capital (*r* = 0.58, *p* < 0.001) and Work Engagement (*r* = 0.51, *p* < 0.001). Psychological Capital and Work Engagement had a strong positive correlation (*r* = 0.65, *p* < 0.001). These findings offer initial backing for the proposed relationships and Psychological Capital’s potential mediation.

**Table 2:** Bivariate correlations among study variables (*N* = 627).

Variable	1	2	3	4
1. Authenticity (AS)	–			
2. Teacher professional identity (TPIS)	0.45**	–		
3. Psychological capital (PCQ-24)	0.52**	0.58**	–	
4. Work engagement (UWES-17)	0.48**	0.51**	0.65**	–

AS, Authenticity Scale; TPIS, Teachers’ Professional Identity Scale; PCQ-24, Psychological Capital Questionnaire-24; UWES-17, Utrecht Work Engagement Scale-17. **p < 0.001.

These correlation patterns reflect the cultural logic of China’s educational context. The strong professional identity-engagement link (*r* = 0.51) aligns with Confucian role-based motivation, where identity provides a primary anchor for teacher commitment (Sun et al., 2022a; Wei, 2008). The PsyCap-engagement link (*r* = 0.65) is consistent with prior meta-analytic findings (Avey et al., 2011), yet in China this likely reflects psychological resources sustained through collective belonging and social duty rather than purely individual agency (Guo et al., 2022; Wernsing, 2014). The moderate authenticity-engagement correlation (*r* = 0.48) suggests that in China’s collectivist, high-accountability system, the pathway from internal value-alignment to visible work energy is less direct than Western studies have found (Cranton and Carusetta, 2004a, 2004b; Wood et al., 2008). Teachers may experience authenticity as a moral obligation tied to role fulfillment (Cheng, Ze) rather than individual self-expression, requiring negotiation of organizational constraints before translating into engagement (Ball, 2003; Ramezanzadeh et al., 2017). The stronger identity-PsyCap correlation (*r* = 0.58) versus authenticity-PsyCap (*r* = 0.52) further supports the Confucian emphasis on role-based self-definition, where identity is forged through relational obligations to students, parents, and the institution (Trent, 2010). These patterns provide initial support for our mediation model while hinting at cultural contingencies explored in the qualitative phase.

#### 4.1.2 Testing the hypotheses

Structural Equation Modeling (SEM) was used to test the hypothesized model with AMOS software (Version 26). The model proposed a mediation framework where Psychological Capital mediates the relationships between Authenticity and Teacher Professional Identity with Work Engagement. It hypothesized direct and indirect effects, with indirect paths through Psychological Capital. The model was evaluated for overall data fit, and path significance and strength were examined.

Prior to testing the structural model, a confirmatory factor analysis (CFA) was conducted to validate the measurement model and assess the latent constructs’ validity. The measurement model included the four latent variables: Authenticity (three indicators: Authentic Living, Self-Alienation, Acceptance of External Influence), Teacher Professional Identity (four indicators: occupational values, role value, sense of belonging, professional behavior inclination), Psychological Capital (four indicators: Self-Efficacy, Hope, Resilience, Optimism), and Work Engagement (three indicators: Vigor, Dedication, Absorption). Factor loadings were generally strong, ranging from 0.72 to 0.89 across constructs, exceeding the 0.70 threshold for acceptable item retention. Composite reliability (CR) values ranged from 0.82 to 0.91, and average variance extracted (AVE) values were between 0.56 and 0.68, supporting convergent validity. Discriminant validity was confirmed as AVE for each construct exceeded squared correlations with others (Fornell and Larcker, 1981). The measurement model fit was adequate: *χ*^2^ (143) = 298.76, *p* < 0.001; CFI = 0.96; TLI = 0.95; RMSEA = 0.052 [90% CI: 0.045, 0.059]; SRMR = 0.039 (Hu and Bentler, 1999). With the measurement model validated, the structural model was then examined.

The mediation model showed strong fit. The *χ*^2^ statistic was significant, *χ*^2^ (149) = 325.42, *p* < 0.001, but interpreted carefully due to its sensitivity to large samples (over 200 cases; Kline, 2016). Focus shifted to less affected indices. The Comparative Fit Index (CFI = 0.95) and Tucker-Lewis Index (TLI = 0.94) surpassed the 0.90 threshold (Hu and Bentler, 1999), showing excellent incremental fit and improvement over a null model. For absolute fit, the Root Mean Square Error of Approximation (RMSEA = 0.055 [90% CI: 0.048, 0.062]) fell below 0.06, and the Standardized Root Mean Square Residual (SRMR = 0.042) was under 0.08, indicating good fit and close match to observed data (Byrne, 2010). These indices together confirmed the model’s strong alignment with the data, supporting path analysis. Table 3 summarizes the fit indices.

**Table 3:** Model fit indices for the hypothesized mediation model (*N* = 627).

Fit index	Value	Recommended level	Interpretation
*χ*^2^ (df)	325.42 (149)	*p* > 0.05 (ideally non-significant)	Sensitive to sample size
*p*-value	<0.001	>0.05	Significant (sample size effect)
CFI	0.95	≥ 0.90	Excellent fit
TLI	0.94	≥ 0.90	Excellent fit
RMSEA [90% CI]	0.055 [0.048, 0.062]	≤ 0.06	Good fit
SRMR	0.042	≤ 0.08	Good fit

*χ*^2^, Chi-square statistic; df, degrees of freedom; CFI, Comparative Fit Index; TLI, Tucker-Lewis Index; RMSEA, Root Mean Square Error of Approximation; SRMR, Standardized Root Mean Square Residual.

**Figure 1:**
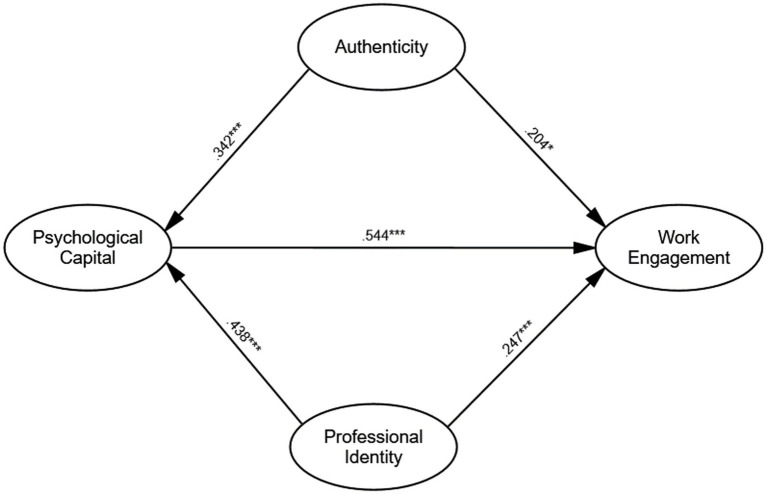
Structural equation model of authenticity and teacher professional identity on work engagement, mediated by psychological capital.

Path analysis in the SEM framework identified significant direct and indirect effects that largely supported the hypothesized model (see Figure 1). Authenticity had a significant positive direct effect on Psychological Capital (*β* = 0.342, *SE* = 0.04, *p* < 0.001), meaning a one standard deviation rise in Authenticity predicts a 0.34 standard deviation increase in Psychological Capital, with other variables held constant. Authenticity also showed a significant positive direct effect on Work Engagement (*β* = 0.204, *SE* = 0.06, *p* < 0.05), pointing to a direct link from authenticity to higher engagement. Teacher Professional Identity displayed significant positive direct effects on both Psychological Capital (*β* = 0.438, *SE* = 0.05, *p* < 0.001) and Work Engagement (*β* = 0.247, *SE* = 0.05, *p* < 0.001). The path from Teacher Professional Identity to PsyCap was notably stronger than from Authenticity to PsyCap, suggesting professional identity has a greater impact on psychological resources in this group. Psychological Capital had a strong positive direct effect on Work Engagement (*β* = 0.544, *SE* = 0.04, *p* < 0.001), the largest coefficient in the model, highlighting PsyCap’s key role in promoting teacher engagement. Table 4 details the path coefficients, including direct, indirect, and total effects. These results confirm Hypotheses 1–3: Hypothesis 1 is supported by the direct path from authenticity to work engagement (*β* = 0.204, *p* < 0.05) and their positive correlation (*r* = 0.48, *p* < 0.001); Hypothesis 2 is supported by the direct path from professional identity to work engagement (*β* = 0.247, *p* < 0.001) and correlation (*r* = 0.51, *p* < 0.001); Hypothesis 3 is supported by the direct path from PsyCap to work engagement (*β* = 0.544, *p* < 0.001) and correlation (*r* = 0.65, *p* < 0.001).

**Table 4:** Standardized path coefficients for the hypothesized mediation model (*N* = 627).

Path	Standardized coefficient (*β*)	SE	*p*-value	95% confidence interval	Effect type
Authenticity → Psychological capital	0.342	0.04	< 0.001	[0.26, 0.42]	Direct
Authenticity → Work engagement	0.204	0.06	<0.05	[0.08, 0.33]	Direct
Teacher professional identity → psychological capital	0.438	0.05	<0.001	[0.34, 0.54]	Direct
Teacher professional identity → work engagement	0.247	0.05	<0.001	[0.15, 0.35]	Direct
Psychological capital → work engagement	0.544	0.04	<0.001	[0.46, 0.63]	Direct
Authenticity → psychological capital → work engagement	0.186	0.03	<0.001	[0.13, 0.24]	Indirect
Teacher professional identity → psychological capital → work engagement	0.238	0.03	<0.001	[0.18, 0.30]	Indirect
Authenticity → work engagement (total effect)	0.390	–	<0.001	–	Total
Teacher professional identity → work engagement (total effect)	0.485	–	<0.001	–	Total

SE, Standard Error. Confidence Intervals (CI) are bias-corrected and accelerated from bootstrapping (5,000 resamples).

To test the mediating role of Psychological Capital formally, indirect effects were measured and assessed. Bootstrapping (5,000 resamples), a non-parametric method robust to normality violations, evaluated significance and provided bias-corrected, accelerated 95% confidence intervals. Results showed significant indirect effects of both Authenticity and Teacher Professional Identity on Work Engagement via Psychological Capital.

The indirect effect of Authenticity on Work Engagement through Psychological Capital was significant (*β* = 0.186, *SE* = 0.03, *p* < 0.001, 95% CI [0.13, 0.24]), as the confidence interval excluded zero, offering clear mediation evidence. The indirect effect of Teacher Professional Identity on Work Engagement via Psychological Capital was also significant (*β* = 0.238, *SE* = 0.03, *p* < 0.001, 95% CI [0.18, 0.30]), supporting PsyCap’s mediation. Comparing direct and indirect effect sizes indicates partial mediation, with both paths contributing to the relationships. These indirect effects strongly back the proposed mediation by Psychological Capital. As Table 4 displays, Authenticity’s total effect on Work Engagement is 0.390, split into a direct effect of 0.204 and indirect effect (via PsyCap) of 0.19. Teacher Professional Identity’s total effect is 0.485, with a direct effect of 0.247 and indirect effect of 0.238. These mediation results support Hypotheses 4 and 5, confirming partial mediation by PsyCap in the relationships of authenticity and professional identity with work engagement.

The model explained substantial variance in Psychological Capital and Work Engagement. Authenticity and Teacher Professional Identity accounted for 42% of Psychological Capital’s variance (*R^2^* = 0.42). Authenticity, Teacher Professional Identity, and Psychological Capital explained 58% of Work Engagement’s variance (*R^2^* = 0.58). In behavioral and social sciences, these *R^2^* values fall in the moderate to large range (Cohen, 1988), indicating solid predictive strength. Unexplained variance suggests that organizational factors such as leadership, school climate, and policy context also play significant roles—a point addressed in the qualitative phase.

The path coefficients in Table 4 reveal a hierarchy of influences reflecting the cultural logic of the Chinese context. PsyCap emerged as the strongest direct predictor of engagement (*β* = 0.544), consistent with meta-analytic evidence (Avey et al., 2011; Luthans et al., 2015) and recent Chinese studies (Guo et al., 2022; Ma, 2023). Professional identity showed a stronger effect on PsyCap (*β* = 0.438) than authenticity (*β* = 0.342), suggesting that role-based identity—grounded in Confucian relational obligations—provides a more powerful foundation for building hope, efficacy, and resilience than internal value-alignment (Trent, 2010; Wei, 2008). The direct path from authenticity to engagement (*β* = 0.204, p < 0.05), while significant, is comparatively modest, indicating that authenticity in China’s collectivist, high-accountability system may require negotiation within relational and institutional constraints before translating into visible work energy (Cranton and Carusetta, 2004a, 2004b; Ramezanzadeh et al., 2017)—a contingency explored later through the case of Mr. Wu.

The significant indirect effects confirm PsyCap as a key mediating mechanism (Bakker and Demerouti, 2007), with partial mediation indicating two parallel pathways: direct influences of authenticity and identity on engagement, and indirect influences through PsyCap. The larger indirect effect of professional identity through PsyCap (*β* = 0.238) versus authenticity (*β* = 0.186) aligns with the cultural logic that professional identity—embedded in social relationships and institutional expectations—is a more potent resource for building psychological resources than the internal, individualistic construct of authenticity (Sun et al., 2022b).

These findings extend prior research in several ways. While Western studies emphasize authenticity as a direct driver of engagement (Cranton and Carusetta, 2004a, 2004b), our results suggest that in China, authenticity’s effects are partially channeled through PsyCap and are weaker than those of professional identity, supporting cross-cultural critiques that Western models may overestimate the universal applicability of individualistic constructs (Hofstede, 2001; Wernsing, 2014). The mediation role of PsyCap is consistent with Guo et al. (2022) and Ma (2023), but our study extends these findings by simultaneously examining two distinct antecedents and demonstrating their differential contributions. The R^2^ value of 0.58 is comparable to or exceeds those reported in similar studies (e.g., Guo et al., 2022; Sun et al., 2022a), yet the unexplained variance underscores the need for future research incorporating organizational and contextual variables.

#### 4.1.3 Summary of quantitative findings in context

Three key findings emerge with significant contextual implications. First, professional identity predicts work engagement more strongly than authenticity, reflecting the Confucian emphasis on role-based self-definition (Wei, 2008). Second, PsyCap robustly mediates both relationships, functioning as a psychological conduit that translates internal resources into visible work energy (Luthans et al., 2015). Third, the model explains substantial variance in engagement (R^2^ = 0.58), yet leaves room for unmeasured organizational factors such as climate, leadership, and policy context.

These findings must be interpreted against China’s evolving educational landscape. The “Double Reduction” reforms have intensified demands for “Quality Education” (Su Zhi Jiao Yu) while high-stakes accountability pressures persist (Sun et al., 2022a). In this context, professional identity may serve as a stabilizing anchor buffering against reform-induced uncertainty, while authenticity may be more fragile where performativity and digital surveillance constrain value expression (Ball, 2003; Lasky, 2005). The strong mediating role of PsyCap suggests that interventions building self-efficacy, hope, resilience, and optimism could effectively sustain engagement amid systemic pressures. However, the qualitative phase below reveals that these relationships are not deterministic—they are deeply moderated by organizational alignment, career stage, and identity pluralism, factors the statistical model cannot fully capture.

### 4.2 Qualitative results

The qualitative phase involved thematic analysis of semi-structured interviews with 26 teachers. It sought to offer deeper, context-specific insights into the quantitative relationships by first identifying common themes that corroborated the model, and second, by analyzing critical cases that explained its boundaries.

#### 4.2.1 Authenticity as congruence and moral compass

Teachers viewed authenticity as matching inner values to professional actions, guided by a “moral compass” in teaching. This connects to quantitative data showing authenticity’s positive ties to psychological capital and work engagement.

##### 4.2.1.1 Value-driven practice

Teachers often saw authenticity as value-based actions centered on care, fairness, and student growth. Ms. Chen, a secondary language teacher, said: “For me, being authentic means that what I do in the classroom, how I interact with students, it all comes from what I truly believe is right and important for them. It’s not just following the textbook or school rules, but really thinking, ‘Is this the best way to help this child learn and grow?’ “(Ms. Chen, Secondary, 12 years). She stressed its student focus: “It’s about seeing each student as an individual, understanding their unique needs, and tailoring my approach to truly meet them where they are, not just delivering a standardized lesson” (Ms. Chen, Secondary, 12 years). This matches quantitative links between high authenticity and stronger engagement, implying value-aligned practice builds purpose and work energy. Rooted in personal beliefs, this purpose drives engagement for authenticity-focused teachers.

##### 4.2.1.2 Principled decision-making

Authenticity showed in firm choices amid pressures. Mr. Li, a primary math teacher, noted: “There are times when the pressure to just focus on test scores is immense. But if I truly believe that a child needs more time, or a different approach, I have to stand by that, even if it’s not the ‘easy’ or ‘popular’ choice. That’s what being true to myself as a teacher means” (Mr. Li, Primary, 8 years). He gave an example: “Last semester, a student was really struggling with fractions. The standardized tests were looming, and the pressure was on to move on. But I knew he just needed more individualized attention. So I spent extra time with him, even though it meant falling slightly behind the planned curriculum. It was the right thing to do, even if it wasn’t what everyone else was doing” (Mr. Li, Primary, 8 years). This echoes quantitative evidence that authenticity boosts psychological capital, as principled actions in tough spots enhance self-efficacy and resilience. Requiring bravery, this approach ties to robust psychological tools for handling job demands.

##### 4.2.1.3 Emotional honesty and vulnerability

Authenticity included open emotions and vulnerability with students and peers. Ms. Wang, a vocational engineering instructor, shared: “In the past, I tried to be this perfect, unshakeable figure in the classroom. But I realized that showing my own humanity, admitting when I do not know something, or sharing my own struggles sometimes, actually builds stronger connections with students. It’s about being real, not just ‘performing’ the role of a teacher” (Ms. Wang, Vocational, 15 years). She noted benefits: “When I’m honest about my own learning process, it models for students that it’s okay to not know everything, that learning is a journey, not a destination. It makes me more relatable, and I think it encourages them to be more open and engaged in their own learning. This transition from a “perfect figure” to a “human” one suggests a reduction in the “emotional labor” often required in teaching, where educators feel compelled to suppress genuine feelings to maintain a professional facade. By choosing vulnerability over surface-level performance, teachers may mitigate the exhaustion associated with chronic emotional labor, thereby preserving psychological energy for genuine engagement.

The qualitative accounts of authenticity as value-driven practice, principled decision-making, and emotional vulnerability resonate with the quantitative finding that authenticity positively predicts work engagement (*β* = 0.204, *p* < 0.05). However, they also reveal a social dimension the survey cannot capture: authenticity is not merely an internal state but a relational negotiation. Teachers describe authenticity in terms of student-centered care and moral courage—suggesting that in China, authenticity is experienced as fulfilling social duties (Ze) rather than expressing individual autonomy (Ramezanzadeh et al., 2017; Wei, 2008). This aligns with the Confucian concept of Cheng (sincerity), where inner virtue is verified through outer role performance, and echoes Trent's (2010) finding that Hong Kong teachers linked authenticity to fulfilling responsibilities rather than personal self-expression. These insights help explain why the direct path from authenticity to engagement, while significant, was comparatively modest (*β* = 0.204): in contexts where authenticity must be negotiated within institutional constraints and relational expectations, its translation into visible work energy requires navigating organizational barriers the quantitative model does not capture.

#### 4.2.2 Professional identity as a source of strength and purpose

A solid professional identity emerged as vital for building psychological capital and engagement. Teachers with clear identities saw it as providing strength, purpose, and resilience, backing quantitative positive links among professional identity, psychological capital, and engagement.

##### 4.2.2.1 Sense of Calling and Commitment

Teachers spoke of a profound calling and dedication to student success. Mr. Zhang, a secondary history teacher, said: “Teaching is not just a job for me, it’s who I am. I feel a real responsibility to guide these young people, to help them discover their potential. That sense of purpose keeps me going, even when things are tough” (Mr. Zhang, Secondary, 20 years). He added on its scope: “It’s not just about the immediate lesson or the current academic year. It’s about planting seeds, shaping future citizens, contributing to something bigger than myself. That’s what gives my work meaning.” This ties to quantitative data where stronger identity scores link to higher engagement, as professional purpose spurs dedication and energy. This innate calling serves as a strong inner motivator, supporting engagement through challenges.

##### 4.2.2.2 Professional efficacy and mastery

Strong identity connected to efficacy and classroom mastery. Ms. Liu, a primary science teacher, described: “When I see a student finally ‘get it,’ when I can break down a complex concept and make it understandable, that’s incredibly rewarding. It reinforces my belief in my abilities as a teacher. It makes me feel like I’m good at what I do” (Ms. Liu, Primary, 6 years). She shared an instance: “Recently, I had a student who was really struggling with the concept of photosynthesis. After several different approaches, using hands-on activities and visual aids, suddenly, it clicked. The look on her face, the excitement in her voice – that’s the moment you realize, ‘Yes, I can do this. I can make a difference.’“This matches quantitative correlations between identity and psychological capital, especially self-efficacy. Firm identity boosts confidence in tackling issues and goals. Efficacy from teaching wins forms a core part of both identity and psychological capital.

##### 4.2.2.3 Community and belonging

Belonging in the profession was key to identity. Mr. Gao, a vocational culinary teacher, explained: “Knowing that I’m part of a larger community of educators, people who understand the challenges and rewards of this profession, that’s really important. Sharing experiences, collaborating with colleagues, it prevents you from feeling isolated and burnt out” (Mr. Gao, Vocational, 10 years). He pointed to gains: “Just knowing there are others facing similar struggles, sharing lesson ideas, getting advice from experienced colleagues – it makes a huge difference. It’s like having a support system built right into your profession.” This may aid psychological capital indirectly, like resilience and optimism, via support networks, explaining mediation findings. Identity’s communal side proves essential for well-being and lasting engagement.

The qualitative themes of calling, efficacy, and community provide contextual depth to the finding that professional identity is the strongest predictor of engagement (*β* = 0.247) and PsyCap (*β* = 0.438). Teachers’ narratives reveal that professional identity is not merely a cognitive self-concept but an embodied sense of belonging to a moral community. The emphasis on calling echoes the Confucian ideal of the scholar-teacher (xue zhe), where identity is inseparable from moral cultivation (Wei, 2008). The theme of community belonging highlights the collectivist dimension: Chinese teachers derive psychological resources from embeddedness in supportive professional networks (Klassen et al., 2012), explaining why professional identity exerts such a strong effect on PsyCap—it provides both a clear role framework and a social infrastructure for building hope, efficacy, and resilience. Identity conflicts also emerge as a concern, aligning with findings that they are a significant source of burnout among Chinese teachers (Sun et al., 2022b; Van Veen and Sleegers, 2006).

#### 4.2.3 Psychological capital as a mediator of engagement

This theme tackles quantitative evidence of psychological capital mediating authenticity and identity with engagement. Data showed how self-efficacy, hope, resilience, and optimism in psychological capital turn authenticity and identity into greater engagement.

##### 4.2.3.1 Self-efficacy as a resource for challenge navigation

Teachers drew on self-efficacy to tackle challenges and sustain engagement. Ms. Lin, a secondary English teacher, recounted: “When I face a class that’s particularly challenging, with behavior issues or learning gaps, my initial reaction might be frustration. But then I remind myself, ‘I’ve handled tough situations before, I have strategies that work.’ That confidence helps me approach the problem proactively, rather than feeling overwhelmed and disengaging” (Ms. Lin, Secondary, 14 years). She added: “It’s like having a toolbox of strategies and knowing how to use them. When I feel efficacious, I’m more willing to experiment, to try different approaches until I find what works. It turns challenges into puzzles to be solved, rather than roadblocks.” This backs quantitative mediation, as authenticity and identity foster PsyCap, enabling self-efficacy for ongoing engagement. Self-efficacy stands as a vital PsyCap element, empowering proactive challenge handling.

##### 4.2.3.2 Hope and goal-directed energy

Hope was key for long-term engagement, aiding goal paths and motivation. Mr. Chen, a primary art teacher, shared: “Teaching can be draining, especially when you do not see immediate results. But I always hold onto hope – hope that my efforts will eventually make a difference, hope that I can find new ways to reach struggling students. That hope fuels my dedication and vigor” (Mr. Chen, Primary, 5 years). He noted persistence: “There are days when you feel like you are not getting through to anyone. But I have to believe that progress is being made, even if it’s not immediately visible. That hope keeps me invested, keeps me searching for new ways to connect with students and inspire them.” This shows PsyCap, via hope, mediating base factors to dedication in engagement. Hope acts as forward-looking motivation, upholding commitment amid slow progress.

##### 4.2.3.3 Resilience in the face of setbacks

Resilience was crucial for engagement in teaching’s demands. Ms. Huang, a vocational nursing instructor, explained: “Setbacks are inevitable in teaching – a lesson that bombs, a student who’s resistant to learning, negative feedback. But I’ve learned to see these as learning opportunities, not personal failures. I recover quickly, adapt, and keep going. That resilience is crucial for staying engaged long-term” (Ms. Huang, Vocational, 18 years). She gave an example: “Last year, I received some really critical feedback on my teaching from a student evaluation. Initially, it stung. But instead of getting discouraged, I used it as a chance to reflect, to identify areas for improvement. I talked to mentors, revised my lesson plans, and came back stronger. That ability to bounce back is essential in this profession.” This supports PsyCap mediation, as resilience in PsyCap helps sustain engagement despite stress. Resilience serves as a protective tool, letting teachers endure issues and keep engaged.

##### 4.2.3.4 Optimism and positive outlook

Optimism contributed to engaging work experiences. Mr. Wang, a secondary physics teacher, noted: “I try to focus on the positive aspects of teaching – the moments of connection with students, the progress they make, the chance to shape young minds. Even on tough days, I believe things will get better. That optimistic outlook makes the job much more enjoyable and engaging.” (Mr. Wang, Secondary, 11 years). He stressed its active side: “It’s not just about passively hoping for the best. It’s about actively looking for the good, celebrating small victories, and maintaining a belief in the potential for positive change. That positive mindset makes all the difference in how I approach my work and interact with my students.” Fostered by PsyCap, this framing clarifies mediation, as PsyCap promotes positivity beyond buffering negatives. Optimism is proactive cognition enhancing work experience and engagement.

The qualitative accounts of self-efficacy, hope, resilience, and optimism provide compelling evidence for PsyCap’s mediating role, consistent with the finding that PsyCap is the strongest direct predictor of engagement (*β* = 0.544). Teachers’ narratives reveal how psychological resources function as a translational mechanism converting authenticity and identity into sustained work energy. Importantly, the expression of these resources in China is culturally shaped: resilience is described not merely as individual “bouncing back” but as drawing strength from collegial support, echoing Wernsing's (2014) observation that PsyCap components may be grounded more in social ties than individualistic agency in collectivist societies. Optimism is framed as a belief in positive change through dedicated effort, aligning with the Confucian value of perseverance (Wei, 2008). These cultural nuances explain why PsyCap is such a powerful mediator in the Chinese context—it bridges the individual and collective, translating personal resources into socially-embedded work energy.

#### 4.2.4 Explaining the “exceptions”: a critical case analysis

While the thematic analysis (4.2.1–4.2.3) largely confirmed the SEM model, the critical case analysis revealed why these relationships are not deterministic. Our holistic analysis of five “deviant” cases identified specific contextual and personal factors that mediate or even reverse the hypothesized paths. We present three such cases below, selected for their representativeness in illustrating the key boundaries of the model.

Case 1: The “Conflicted Authentic” Teacher (High Authenticity, Low Engagement). Mr. Wu, a 15-year veteran high school teacher, scored in the 90th percentile for Authenticity but in the 20th for Work Engagement. His case challenges the direct positive path from authenticity to engagement. His interview revealed that his authenticity was, in fact, the *source* of his disengagement. Mr. Wu described his core values as “student-centered” and “creativity-focused.” However, he depicted his school environment as “rigidly conformist” and “obsessed with test scores.” He explained: “I *am* authentic. I tell my principal what I think. I refuse to just ‘teach to the test.’ And for this, I am punished. My ideas are ignored, I am given the worst schedules. Being true to myself here is exhausting. It makes me want to give up, not ‘engage’ more” (Mr. Wu, Secondary, 15 years). Mr. Wu’s case suggests that authenticity in a non-aligned or hostile environment, rather than fostering engagement, can lead to conflict, exhaustion, and burnout. This highlights the role of organizational context as a critical moderator not captured in the quantitative model. Mr. Wu’s case refines the JD-R model by illustrating that “personal resources” are not inherently positive; in a misaligned organizational context, the drive for authenticity can transform from a resource into a significant psychological “demand.” This challenges the SDT assumption that autonomy always leads to flourishing, suggesting instead that when autonomy (acting on one’s values) results in systemic punishment, it leads to resource depletion.

Case 2: The “Engaged but Inauthentic” Teacher (Low Authenticity, High Engagement). Ms. Fang, a 4-year primary teacher, scored in the 25th percentile for Authenticity (specifically high on the “Acceptance of External Influence” subscale) but in the 85th for Work Engagement. Her case contradicts the model’s premise that authenticity is a necessary antecedent. Her narrative showed a different pathway to engagement. When asked about her values, Ms. Fang stated: “Honestly, I’m still figuring that out. I mostly just try to do what my mentor teacher and the curriculum require.” Her low authenticity score reflected this. However, her high engagement (vigor and dedication) was palpable. She explained her motivation: “I just love the *craft* of it. I love seeing a lesson plan work perfectly. I love the ‘Aha!’ moment from a student. and my teaching team is fantastic. We are very close”(Ms. Fang, Primary, 4 years). Ms. Fang’s case suggests that engagement, particularly for early-career teachers, may be driven less by a deep, internal value-congruence and more by social belonging and the psychological rewards of skill mastery and efficacy—factors related to, but distinct from, authenticity. Ms. Fang’s narrative suggests that for novice teachers, external validation—derived from successful lesson execution and peer approval—carries significantly more weight than internal authenticity. Her engagement was fueled by “scaffolded efficacy” rather than value-congruence. This refines SDT by suggesting that in the induction phase, “identified regulation” (valuing the craft) and social belonging can bypass the need for “intrinsic” authenticity to sustain high vigor and dedication.

Case 3: The “Resilient but Detached” Teacher (High PsyCap, Low Professional Identity). Mr. Huang, a vocational teacher with 10 years of experience, scored in the 95th percentile for PsyCap (especially resilience and self-efficacy) but in the lowest 15th for Professional Identity. This case challenges the strong link between identity and PsyCap. Mr. Huang’s interview was characterized by high confidence and a ‘can-do’ attitude. “Problems? They’re just puzzles. A difficult student is a puzzle. Bad admin is a puzzle. I can handle it,” he noted. Yet, he explicitly rejected the “teacher” label: “I do not really see myself as a ‘teacher’ in that. communal sense. I’m an engineer who teaches. My loyalty is to my subject, to industry standards, not to the ‘teaching profession.’ I do not go to the school social events” (Mr. Huang, Vocational, 10 years). Mr. Huang’s case reveals that the resources of PsyCap can be drawn from an alternative, non-professional identity (e.g., a “subject-matter expert” identity). This suggests the model’s focus on *professional* identity as the sole source of such resources may be too narrow. Mr. Huang’s case extends the JD-R framework by demonstrating that personal resources like resilience and self-efficacy are not exclusively anchored to a single professional identity. It suggests that a “disciplinary identity” can act as a substitute resource, providing the psychological strength necessary to remain engaged even when the teacher feels alienated from the broader pedagogical community.

These cases collectively demonstrate that the relationships in the SEM model, while statistically significant, are not universal. They are deeply embedded in, and can be overridden by, an individual’s specific organizational context, career stage, and alternative sources of personal identity.

The three critical cases reveal the boundaries of the quantitative model and highlight social contingencies shaping teacher motivation. Mr. Wu’s case demonstrates that authenticity misaligned with organizational culture can become burnout-inducing, reflecting the “Harmony-Authenticity Paradox” where relational harmony (He) can punish authentic dissent (Ball, 2003; Lasky, 2005). Ms. Fang’s case challenges the assumption that authenticity is a necessary antecedent of engagement, particularly for novice teachers whose engagement may be driven by social belonging and pedagogical mastery—scaffolding that supports identity development (Beauchamp and Thomas, 2009; Pillen et al., 2013). Mr. Huang’s case reveals that psychological resources can be sustained through alternative identities, particularly disciplinary expertise, providing a buffer against institutional pressures—a finding with particular relevance for China’s vocational education sector, where teachers often have strong industry backgrounds but weaker identification with the teaching profession (Hoyle and John, 1995; Olsen, 2008). Collectively, these cases demonstrate that the SEM relationships, while statistically robust, are not deterministic. They are deeply shaped by organizational context, career stage, and identity pluralism—factors the quantitative model could not capture, revealing that teacher motivation in China is embedded in complex social, institutional, and cultural dynamics (Bakker and Demerouti, 2007; Carmona-Halty et al., 2019).

#### 4.2.5 Synthesis of qualitative findings in context

The qualitative phase serves two complementary purposes: corroborating the quantitative model by revealing mechanisms through which authenticity, identity, and PsyCap operate, and extending it by exposing boundaries and contingencies. The thematic analysis confirms that authenticity provides a moral foundation, identity offers a social framework, and PsyCap acts as an agentic engine translating these resources into visible work energy. However, the critical case analysis reveals these pathways are not universal—engagement is contingent on organizational alignment, career stage, and identity pluralism, factors Western-derived models often treat as secondary.

The qualitative findings refine the quantitative model in three ways. First, authenticity’s effects are conditional: in misaligned organizational contexts, authenticity can deplete rather than build psychological resources, extending the JD-R model by showing personal resources are not inherently positive (Bakker and Demerouti, 2007). Second, multiple pathways to engagement exist, including social and developmental routes that do not require fully formed authenticity or professional identity, supporting a more pluralistic understanding of teacher motivation (Akkerman and Meijer, 2011). Third, psychological resources can be sustained through alternative identities—particularly disciplinary expertise—that provide a buffer against institutional pressures. Theoretically, these findings extend the JD-R model by showing personal resources are context-dependent and identity pluralism offers alternative pathways to PsyCap. Practically, they suggest interventions must attend to organizational conditions and career-stage factors shaping how psychological resources translate into sustained work energy—a contribution enabled by integrating quantitative breadth with qualitative depth (Creswell and Plano Clark, 2017).

## 5 Discussion

This mixed-methods study provides a detailed understanding of the relationships among teacher authenticity, professional identity, psychological capital (PsyCap), and work engagement in the context of mainland China’s education system. The quantitative SEM results established a robust statistical model, which was then elaborated upon by a qualitative phase. This dual-phase approach allowed the research to first identify the key general associations among these factors and, second, to use critical case analysis to explain important, nuanced exceptions and contextual factors that define the model’s boundaries. These integrated results add to the growing body of work on teacher well-being and performance, especially in Chinese settings, and point to useful directions for theory and practice.

The quantitative results and the foundational thematic analysis provide clear evidence for authenticity’s positive effects. Specifically, the SEM results suggest that in China, authenticity operates as a “moral anchor.” While Western teachers might seek authenticity for personal “flourishing,” our findings indicate that for Chinese teachers, aligning actions with core values serves as a path to fulfilling social duties (*yiwu*). This cultural alignment reduces the cognitive dissonance between a teacher’s personal ethics and their professional persona, thereby preserving psychological resources (Trent, 2010). The SEM model’s significant positive paths (Table 4) align with the qualitative theme of authenticity as a “moral compass” (Section 4.2.1). Teachers who align actions with core values report stronger PsyCap and engagement (Cranton and Carusetta, 2004a, 2004b; Ramezanzadeh et al., 2017). In China’s cultural environment, this alignment may be particularly potent, as Confucian values of moral self-cultivation and responsibility frame authenticity as a path to fulfilling social duties, thereby reducing conflict and boosting psychological resources.

However, our critical case analysis reveals this relationship is not absolute. The case of Mr. Wu (“The Conflicted Authentic”) highlights the “Harmony-Authenticity Paradox” unique to the Chinese workplace (Hofstede, 2001; Wei, 2008). In the current educational climate, where digital monitoring and “social credit” style evaluations are common, high levels of individual authenticity can become a significant professional liability. Mr. Wu’s experience demonstrates that when a teacher’s “moral sincerity” leads them to challenge a “test-obsessed” culture, the resulting friction is not merely social but systemic (Ball, 2003; Lasky, 2005). In a culture that prioritizes *He* (harmony), dissent is often framed as a failure of professional duty (*Yiwu*). For Mr. Wu, the act of being authentic required a level of “emotional labor” that exceeded his psychological capital (Luthans et al., 2015). While authenticity typically reduces dissonance, Mr. Wu’s narrative reveals that when personal pedagogical philosophy directly contradicts administrative mandates, “surface acting”—the suppression of one’s true beliefs to maintain institutional harmony—becomes a constant requirement (Sutton, 2020; Van Veen and Sleegers, 2006). This chronic emotional labor transforms authenticity from a motivator into a psychological burden. Specifically, authenticity shifts from a resource to a drain under conditions of “Value Incongruence,” where the teacher must choose between “moral betrayal” (compliance) and “professional martyrdom” (dissent). In such misaligned environments, the psychological cost of maintaining a “true self” leads to rapid resource depletion and marginalization, eventually resulting in burnout. Therefore, the positive effect of authenticity on engagement in China is heavily moderated by organizational “face” (*Mianzi*) and the degree of value-alignment between the individual and the collective. This suggests that the contemporary “cost” of authenticity is higher than in Western contexts, as the Chinese teacher must navigate a triple burden: personal integrity, relational harmony, and systemic compliance. His experience suggests that the act of being authentic, when in direct conflict with school culture, drains engagement rather than fueling it (Ball, 2003).

Similarly, the strong positive link between professional identity and PsyCap/engagement in the SEM model was broadly supported by themes of “calling” and “community” (Section 4.2.2). A strong identity appears to be a key resource, consistent with prior work (Day and Gu, 2007; Sun et al., 2022a). Yet, the critical cases again challenge the simplicity of this link. The case of Mr. Huang (“The Resilient but Detached”) demonstrates that professional identity, as measured, may not be the only source of PsyCap. Mr. Huang’s high resilience and efficacy were drawn from an alternative, “subject-matter expert” identity (“an engineer who teaches”). While he felt little connection to the broader “teaching profession,” his deep identification with his disciplinary field provided him with the “expert efficacy” needed to remain engaged. This finding aligns with the “identity constellation” framework (Akkerman and Meijer, 2011) and suggests that our quantitative model’s focus on a single professional identity is too narrow. For teachers in specialized fields, a strong disciplinary identity may act as a protective buffer, sustaining PsyCap even when the teacher feels alienated from the pedagogical environment.

Furthermore, the case of Ms. Fang (“The Engaged but Inauthentic”) challenges the model’s implied sequence. As a novice teacher, she reported low authenticity and a developing identity but high engagement. Her narrative suggests that, for early-career teachers, engagement may be driven by more immediate factors like social belonging in a team and the mastery of teaching craft. This finding suggests that the relationship between these constructs may be non-recursive over the professional lifespan. While the SEM model captures the “steady-state” logic dominant in the general sample—where established internal values drive external engagement—Ms. Fang’s case reveals a “reverse-causal scaffolding” effect characteristic of the induction phase (Flores and Day, 2006; Pillen et al., 2013). In this developmental context, high engagement does not follow identity; rather, it precedes and facilitates it. By actively engaging in the social and technical practices of the classroom, novice teachers accumulate the “experiential data” necessary to eventually crystallize a stable professional identity and sense of authenticity (Beauchamp and Thomas, 2009; Olsen, 2008). Thus, the qualitative data do not refute the SEM’s linear paths but rather identify career stage as a critical moderator that determines the directionality of the model’s entry point.

Finally, the quantitative analysis confirmed PsyCap’s powerful mediating function, which was well-supported by qualitative accounts of teachers using self-efficacy, hope, resilience, and optimism to navigate their work (Section 4.2.3). This finding remains robust (Avey et al., 2011; Guo et al., 2022; Ma, 2023). What the case studies add, however, is a more complex understanding of the antecedents that build this PsyCap. While authenticity and professional identity are clearly two major pathways (Luthans et al., 2007a, 2007b; Zhang et al., 2024), PsyCap can also be sustained via alternative identities (Mr. Huang) or, in the short term, may be less relevant to engagement than factors like skill mastery and team belonging (Ms. Fang). PsyCap’s role as a mediator is clear; where this PsyCap comes from is more complex than the model suggests (Luthans et al., 2015). Overall, this study’s mixed-methods design provides a nuanced model. The quantitative results show the most likely pathway for the average teacher: authenticity and a strong professional identity build psychological capital, which in turn drives work engagement. The qualitative findings, however, move beyond this “average” to explain the reality: these relationships are not deterministic and are deeply moderated by organizational context, career stage, and the specific source of a teacher’s personal and expert identity.

## 6 Theoretical and practical implications

These findings have significant implications for both theory and practice, particularly within the context of Chinese education. Theoretically, this study provides a unique, culturally-grounded model, as the qualitative data suggests that the teacher-perceived “moral compass” of authenticity aligns well with traditional Confucian values of self-cultivation. Furthermore, these findings enrich Self-Determination Theory (SDT) by re-conceptualizing autonomy within collectivist settings. While Western applications often equate autonomy with independence, our data suggests that for Chinese teachers, it is experienced as “internalized duty.” Drawing on the Confucian concept of *Xiu Shen* (self-cultivation), authenticity acts as a bridge that enables teachers to internalize social and professional obligations, transforming external pressures into autonomous motivation. This indicates that in non-Western contexts, SDT is most effective when accounting for how cultural values—such as professional devotion—allow individuals to find volitional depth within collective boundaries. By integrating these nuances, we offer a “Relational-Authenticity Model” for collectivist environments, positing that authenticity is a social negotiation rather than a purely individual possession. As automated systems take over routine instruction, the teacher’s primary value-add is increasingly viewed as their “authentic human presence.” However, our study suggests that the Confucian emphasis on duty and harmony may inadvertently stifle this unique human advantage if school cultures remain rigidly conformist.

Crucially, this research extends the “Personal Resources” category within the Job Demands-Resources (JD-R) model (Bakker and Demerouti, 2007) by positioning authenticity as a foundational resource that fuels the motivational process. Unlike traditional JD-R personal resources (e.g., self-efficacy) which are often treated as stable internal traits, we demonstrate that authenticity is subject to “boundary effects” created by the organizational context. When a teacher’s values are in direct conflict with systemic demands, this personal resource can be depleted, illustrating that the utility of authenticity in the JD-R framework is heavily moderated by Person-Environment (P-E) fit.

The findings move beyond a simplistic “authenticity is good” framework. The case of Mr. Wu introduces a critical contingency, implying that theories on authenticity must incorporate person-environment fit as a key moderator; his experience demonstrates that authenticity in a hostile or misaligned environment may actually be detrimental to well-being. In addition, the findings challenge the singular focus on professional identity as an antecedent. The case of Mr. Huang suggests that teacher motivation theories must be expanded to include “identity pluralism.” For teachers in specialized fields, a “subject-matter expert” identity serves as a potent alternative source of PsyCap. Furthermore, for novice teachers like Ms. Fang, our results highlight the relevance of social socialization theory (Van Maanen and Schein, 1979). In the absence of a fully formed professional identity, external social support and technical scaffolding during the integration phase act as the primary drivers of engagement. These cases also point toward alternative developmental pathways, especially for novice teachers, where engagement driven by social factors and skill mastery may precede the formation of a stable authentic or professional identity.

These theoretical nuances, in turn, lead to more targeted practical insights. While the general advice to “foster authenticity and identity” is valid, our findings demand a more sophisticated approach. For school leaders, the focus must move beyond a “surveillance-based” harmony toward a “pluralistic harmony” that allows for authentic professional disagreement. As a policy priority, school management should consciously avoid “identity homogenization”—the institutional pressure for all faculty to adopt a singular, standardized pedagogical persona. Instead, leaders should allow teachers to maintain their “expert identities” as disciplinary specialists. Recognizing teachers as both pedagogues and subject-matter experts provides a critical moral buffer that sustains PsyCap. To mitigate these tensions, leaders should implement “Value-Alignment Audits”—structured, non-evaluative forums where staff can safely discuss the friction between “Quality Education” (*Su Zhi Jiao Yu*) ideals and high-stakes testing pressures. Additionally, schools can protect high-integrity teachers by adopting “Differentiated Professional Recognition” systems. Moving away from monolithic test-score evaluations to formally recognize “pedagogical innovators” or “well-being advocates” provides a structural “moral buffer,” allowing teachers like Mr. Wu to maintain integrity without facing marginalization. In today’s hyper-monitored environments, leaders must provide concrete “Psychological Safety Zones” where dissent is reframed as professional commitment (*Yiwu*) rather than a disruption of relational harmony (*He*).

Similarly, teacher educators should recognize these multiple pathways to resilience. For modern-era professional development, this means shifting the focus from mere technical skills to “identity resilience”—helping teachers maintain their moral core (*Xin*) while navigating the intensifying demands of digital and social duty. Professional development could be strengthened by acknowledging that for some teachers, like Mr. Huang, “reconnecting with your subject-matter passion” may be a more effective intervention for building PsyCap than “reflecting on your professional identity.” Finally, the “engaged but inauthentic” case of Ms. Fang offers clear guidance for induction education. Grounded in socialization theory, early-career support should prioritize fostering immediate social belonging and building concrete pedagogical mastery. This external “scaffolding” is critical during the integration phase, as it provides the necessary environmental support to sustain engagement until a deeper, value-driven authenticity has time to crystallize over the long term.

### 6.1 Limitations and future directions

While this study offers valuable insights, it is important to acknowledge certain limitations. First, the reliance on self-report measures for the quantitative phase introduces the potential for common method bias (CMB). Although statistical checks, such as Harman’s single-factor test, suggested that CMB did not significantly threaten the validity of the current results, these statistical remedies cannot fully replace methodological ones. Therefore, while the bias appears controlled in this sample, future research should prioritize multi-source data validation—such as incorporating supervisor ratings or student evaluations—to provide a more objective assessment of teacher behavior. Furthermore, the cross-sectional nature of the quantitative data limits the ability to draw definitive conclusions about causality.

The qualitative findings point to several advanced directions for future research that move beyond these general limitations. Mr. Wu’s case, for example, strongly suggests that future quantitative studies should explicitly test the moderating role of organizational climate—such as perceived value congruence—on the relationship between authenticity and work engagement. Similarly, Mr. Huang’s experience indicates that research should move beyond a monolithic professional identity construct. Building on the “identity constellation” framework discussed in this study, future investigations should differentiate between teachers’ pedagogical, organizational, and disciplinary (subject-matter expert) identities to quantify their unique contributions to PsyCap. The developmental questions raised by Ms. Fang’s case also highlight the need for longitudinal research. Such studies could track novice teachers over time to test alternative causal models, examining whether early-career engagement driven by social and mastery factors might actually predict the later development of professional identity and authenticity. This longitudinal work could also incorporate multi-method approaches, such as classroom observation, to triangulate teachers’ reported authenticity with their enacted practices, providing a more robust account of how internal values translate into classroom behavior within constrained educational systems.

Finally, the shift toward an AI-integrated educational landscape introduces a new frontier for teacher authenticity. Future research should investigate “digital authenticity,” specifically exploring how teachers maintain their moral standards and professional integrity (*Xin*) when navigating the influence of AI algorithms and automated instructional prompts. As algorithmic management becomes more prevalent in Chinese schools, understanding how teachers negotiate the tension between data-driven efficiency and their authentic pedagogical beliefs will be essential for sustaining long-term engagement.

## 7 Conclusion

This study examined how teacher authenticity, professional identity, and psychological capital relate to work engagement among K-12 teachers in mainland China. Using an explanatory sequential mixed-methods design, we tested a mediation model grounded in the Job Demands-Resources framework. Quantitative results confirmed that authenticity and professional identity predict work engagement both directly and indirectly through psychological capital, with professional identity showing stronger effects—consistent with Confucian role-based self-definition. The qualitative critical case analysis extended these findings by revealing when and why these relationships break down. Authenticity can become a psychological burden in misaligned organizational contexts; novice teachers may sustain engagement through social belonging and skill mastery rather than deep value congruence; and disciplinary expertise can substitute for professional identity as a source of psychological resilience. These exceptions demonstrate that the model’s pathways are moderated by organizational culture, career stage, and identity pluralism.

Theoretically, this study contributes to the JD-R model by showing that personal resources are subject to boundary effects shaped by institutional context. It also challenges the universal applicability of Western authenticity models by revealing a “Harmony-Authenticity Paradox” in Confucian-influenced settings, where moral sincerity must be negotiated alongside relational harmony and systemic compliance. Practically, fostering teacher engagement requires attention to organizational conditions that enable authentic practice, recognition of multiple identity sources, and developmental support tailored to career stage. Teacher engagement in China’s high-stakes system thus emerges not simply from personal resources but from the dynamic interplay between individual dispositions and the institutional and cultural contexts in which teachers work. Researchers and practitioners must attend to both to sustain the vigor, dedication, and absorption that define meaningful professional engagement.

## Data Availability

The raw data supporting the conclusions of this article will be made available by the authors, without undue reservation.
